# Roles and challenges of family physicians in Uganda: A qualitative study

**DOI:** 10.4102/phcfm.v11i1.2009

**Published:** 2019-10-29

**Authors:** Innocent K. Besigye, Jude Onyango, Fred Ndoboli, Vincent Hunt, Cynthia Haq, Jane Namatovu

**Affiliations:** 1 Department of Family Medicine, Makerere University, Kampala, Uganda; 2 Department of Family Medicine, The Warren Alpert Medical School, Brown University, United States; 3 Department of Family Medicine and Community Health, The University of Minnesota Medical School, Champlin, Minnesota, United States; 4 Department of Family Medicine, University of California, Irvine, California, United States

**Keywords:** family medicine, family physician, family practice, primary care, roles, challenges

## Abstract

**Background:**

The World Health report (2008), the World Health Assembly (2009) and the Declaration of Astana (2018) acknowledge the significant contribution of family physicians (FPs) in clinical and primary healthcare. Given the lack of resources and low numbers of FPs coupled with the contextual nature of family medicine (FM), the scope of practice of African FPs is likely to differ from that of colleagues in America and Europe. Thus, this study explored the roles of Ugandan FPs and the challenges they face.

**Methods:**

This cross-sectional qualitative study was conducted through in-depth interviews with FPs who are working in Uganda. Participants who work in public and private healthcare systems including non-governmental organisations and in all geographical regions were purposively selected. Interviews were conducted from July 2016 to June 2017. Qualitative thematic content analysis of the transcripts was performed using a framework approach.

**Results:**

The study team identified three and six thematic roles and challenges, respectively, from the interview transcripts. The roles were clinician, leadership and teaching and learning. Challenges included lack of common identity, low numbers of FPs, conflicting roles, unrealistic expectations, poor organisational infrastructure and lack of incentives.

**Conclusion:**

The major roles of FPs in Uganda are similar to those of their counterparts in other parts of the world. Family physicians provide clinical care for patients, including preventive and curative services; providing leadership, management and mentorship to clinical teams; and teaching and learning. However, their roles are exercised differently as a result of lack of proper institutionalisation of FM within the Uganda health system. Family physicians in Uganda have found many opportunities to contribute to healthcare leadership, education and service, but have not yet found a stable niche within the healthcare system.

## Background

The World Health Report, the World Health Assembly and the Declaration of Astana acknowledge the significant contribution and roles of family physicians (FPs) in clinical and primary healthcare.^1,2,3^ Family medicine (FM) training prepares doctors to provide comprehensive primary healthcare to individuals, families and communities throughout their life cycle. In addition, FPs are well prepared to address the clinical and public health problems facing patients, communities and health systems. The World Organisation of Family Doctors (WONCA) Africa region has emphasised the potential contribution of FM to strengthen African health systems and the critical importance of training FPs to meet the healthcare needs of African populations.^4^

The rapid development of FM on the African continent is largely attributed to the realisation of its potential contributions to the performance of health systems by politicians, health managers and academics.^5,6,7,8^ In 2000, FM as a specialty was well known only in South Africa and Nigeria. By 2018, FM had been established in almost all African countries.^9,10,11,12,13,14,15,16^ Those countries without FM training programmes, such as Burundi and South Sudan, are contemplating starting the discipline. However, while most African countries are training FPs, the number of training programmes and graduates remains quite low. Most physicians practising in Africa remain generalists who have not completed postgraduate specialty training programmes.

Most African countries have their FM training programmes based on European and American models. However, the context, organisation and scope of FM practice on the African context differ from that in Europe and America. For example, in Africa, there is a need for FPs to have surgical, procedural and anaesthetic skills because of a widespread lack of surgeons and other specialists in rural areas. Nurses, midwives, clinical officers and other health professionals provide most primary healthcare in Africa. Most African FPs provide secondary care in referral or district hospitals and in urban centres,^17^ though this is not the case for FPs in Europe or the United States. It is therefore likely that the roles of African FPs are different from those of their European and American counterparts. Limited literature exists on the roles and challenges of FPs in Africa. Few studies have documented their roles in provision of clinical services, education, research, leadership, advocacy, community outreach and quality improvement.^18,19,20,21,22,23,24^

There are still two types of generalist physicians in Uganda. The majority are general practitioners (GPs) who have completed undergraduate medical training and a 1-year internship. Ugandan FPs complete undergraduate training and an internship followed by a 3-year postgraduate programme including, additional training in surgery, obstetrics and gynaecology, internal medicine, paediatrics, anaesthesia, psychiatry, district health services, quality improvement, family and community-oriented primary care, research methods and management of health systems. This training aims to produce highly competent FPs who are able to address more than 80% of the health needs of communities and/or populations. Ugandan FPs often work alongside generalists with limited or no postgraduate training. Currently, 70 FPs are registered in Uganda for a population of 40 million people, with only four working in other countries. The roles of Ugandan FPs and the differences between GPs and FPs have not yet been clearly delineated. There is therefore a need to understand the roles of Ugandan FPs and the challenges they face to better determine their place in the national healthcare system. This study explored the roles and challenges of FPs working in Uganda.

## Methods

### Study design

This cross-sectional qualitative study was conducted through in-depth interviews with FPs working in Uganda.

### Setting

The study was conducted in Uganda where FM was first established as a postgraduate specialty in 1989 at Makerere University. Currently, training programmes are located at Makerere and Mbarara universities with approximately 70 graduates by the year 2017 to date. The Ugandan health system consists of the central ministry of health and the district health system (DHS). The ministry of health provides stewardship for the overall national health system and specialised services in regional referral and national referral hospitals. The DHS is responsible for providing primary care through the various health centres (HCs). The HCs are graded from the HC I that is a village health team comprising non-trained community members who link the community with the formal health system through referral of community members whom they think need care; HC II is a dispensary and HC III provides maternal health services in addition to outpatient services to HC IVs and general hospitals that are the referral centres. The Ugandan health policy provides that doctors should work from the level of HCIV upwards. Therefore, FPs work at all levels where policy allows doctors to work.

### Selection of participants

The study team identified FPs working in Uganda to participate in the study. Participants were purposively selected to include FPs working in different geographic regions and in public, private and non-governmental organisation healthcare settings. The selected participants were contacted and invited to participate by email or phone one after another. Interviews were conducted from July 2016 to June 2017. Interviewing was stopped when no new information was being generated from the last three interviews and when it was deemed saturation which has been achieved.^25^

### Data collection

The study team trained two research assistants to conduct in-depth interviews using a semi-structured interview guide (Box 1). Research assistants asked FPs to reflect on their roles and challenges they faced as they carried out their duties in their respective workplaces. Interviews were conducted in English and audiotaped using a digital voice recorder.

BOX 1 Semi-structured interview guide.

**Exploring the roles and challenges of family physicians in Uganda**

1. Can you tell us about yourself?
2. Tell us about your work as a family physician.
3. Briefly describe a typical day in your work or practice.
4. What do you think about the issues facilitating and/or hindering your practice of family medicine?
5. What do you think about your role within the primary healthcare framework of Uganda?
6. What challenges do you face as a family physician in Uganda?
7. In your opinion, what do you think about the appropriate approaches to solve these challenges? (Here, we explored both training and practice approaches to mitigating the challenges.)
8. What do you think are the available opportunities for family physicians in the Ugandan health system?

### Data analysis

The following steps were followed:

- Interviews were recorded, transcribed and numbered. Responses were coded and pooled to protect confidentiality of participants.
- The interviews were transcribed verbatim by the research assistants. The lead author transcribed three of the interviews to gain a better understanding of the participants’ responses.
- The research team read the transcripts and identified themes.
- Codes were developed independently by researchers reading through the transcript line by line, making labels and comments on the margins.
- Each transcript was independently coded by three different members of the research team. This process was completed for the first six transcripts.
- The final set of codes was agreed upon by the research team meeting and discussing the codes and their decisions. The codes were defined, and then grouped into categories to form a working analytical framework.^26,27^
- Each category was assigned a letter abbreviation and each code of a number for easy identification.
- The codes from this framework were then used to index subsequent transcripts.
- Data were summarised by category from each transcript.
- Members of the research team met regularly to discuss impressions and ideas emerging from the data, their individual interpretations of the data and to reach consensus about coding.

### Ethical consideration

Ethical clearance was obtained from the Makerere University School of Medicine Research and Ethics committee (#REC REF 2016-067). Written voluntary informed consent was obtained from participants prior to their interviews.

## Results

A total of 18 interviews were conducted and summarised when the research team agreed that there was no new information emerging and the study had reached saturation. Table 1 presents the description of the study participants.

**TABLE 1:** Description of study participants.

Participant	Age (years)	Gender	Years after graduation	Nature of practice and region
P01	38	Male	05	Clinical, private practice, central
P02	39	Male	01	Clinical and managerial, public, central
P03	48	Male	12	Academic, public, western
P04	45	Male	13	Managerial, Non-Governmental Organisation (NGO), north
P05	43	Male	01	Clinical and managerial, private not for profit, north
P06	47	Male	14	Clinical and managerial, public, western
P07	42	Male	04	Managerial, private not for profit, central
P08	36	Male	02	Clinical and managerial, NGO, central
P09	33	Male	02	Clinical, public, central
P10	45	Male	10	Academic, public, central
P11	45	Male	09	Clinical and managerial, public, central
P12	41	Male	02	Academic, public, central
P13	49	Male	13	Clinical and managerial, public, eastern
P14	48	Female	10	Clinical, private practice, eastern
P15	55	Female	23	Managerial, public, eastern
P16	35	Female	03	Clinical, private, central
P17	35	Male	01	Clinical, public, north
P18	36	Female	02	Clinical, private, western

Three themes were identified regarding the roles, and six themes emerged regarding the challenges faced by FPs working in Uganda. Tables 2 and 3 summarise the themes and sub-themes that emerged from the interviews regarding the roles and challenges, respectively.

**TABLE 2:** Themes and sub-themes for roles.

Themes	Sub-themes
Clinician	Clinical practice settings Holistic care with family orientation Wider scope of practice Referrals
Leader	Management and administration Teamwork and collaboration Quality assurance
Teacher and learner	Educating patients and communities Teaching students, peers and other health professionals Self-education for continuous professional development

**TABLE 3:** Themes and sub-themes for challenges.

Theme	Sub-themes
Lack of identity	Lack of knowledge about FM among patients or public and community Limited knowledge about FM among students and colleagues Limited knowledge about FM within the health system leaders and the ministry of health
Low numbers of family physicians	Improper recruitment Feelings of isolation
Conflicting roles	Heavy clinical workload Clinician and leader Clinician and teacher
Unrealistic expectations	Internal sources External sources
Poor organisational infrastructure	No defined population No place within the health system
Lack of incentives	Promotional hierarchy Low salaries

FM, family medicine.

## Roles of family physicians

### Clinician

Most participants described the provision of clinical services as their main role. Within clinical services, participants described several sub-themes such as clinical practice settings, holistic care with family orientation, their wide scope of practice and making appropriate referrals.

#### Clinical practice settings

Participants described working in a variety of settings to provide clinical care to individuals, families and communities. Family physicians provide clinical services in general outpatient clinics and to in-patients in general, district and referral hospitals and to communities through home visits. The following quotes from participants illustrate these sub-themes:

‘I work like any other doctor within the hospital. I sit and see patients and make clinical decisions.’ (P 06)

‘About 50-60% of my work is clinical. We have a primary care clinic which I run with two other doctors.’ (P 08)

‘I have been involved in taking care of private patients who are on medical insurance and those from NGOs and organisations that have a contract with the facility where I work. My work is mostly clinical.’ (P 16)

It was evident that most FPs cared for patients when they were admitted to hospital wards, although some also played some roles outside the hospital. The roles described included caring for patients within the inpatient and outpatient settings and during outreach activities:

‘I work in a regional referral hospital and I also manage patients within a hospital setting.’ (P 06)

‘I find myself playing some roles within the hospital but I also find myself with some roles outside the hospital. I provide support supervision and also oversee programmatic activities like the expanded programme on immunisation, health management information systems within the region.’ (P 06)

#### Holistic care with family orientation

Most FPs described holistic care to their practice populations. Family physicians viewed their provision of holistic care by applying principles of FM that were inculcated during their training:

‘… looking at families, (providing) family-centered care and being patient-centered most of the time.’ (P 01)

‘The training made me able to handle patients in a holistic approach.’ (P 02)

#### Wider scope of practice

Many participants described their scope of practice as encompassing a continuum of care from the home to community, clinic and hospital and by caring for a variety of patients. Family physicians described caring for patients of all ages, providing emergency surgical and medical care and performing outreach and home visits. Their practice generally includes a focus on prevention and health promotion:

‘My role is to promote basic health care policies like disease prevention, promotion of health, treatment of disease and also rehabilitation.’ (P 05)

‘Indeed I see people of all ages including children, teenagers and a lot of adults.’ (P 01)

‘We have an emergency unit where I see the emergency surgical and medical patients.’ (P 05)

‘I have participated in outreach activities for management of cases.’ (P 06)

‘We do home visits to see where they are living.’ (P 07)

Some participants reflected that they saw patients who had been referred to hospital settings – an indication of some FPs providing secondary clinical care:

‘I see mothers who have been referred from lower level health facilities and operate (on) them if they require caesarean section.’ (P 05)

‘I am primarily involved in patient care specifically in a regional referral hospital.’ (P 06)

‘I also work on issues like infection control, issues related to immunization and maternal and child health services within the hospital setting.’ (P 06)

#### Referrals

Some participants reported making appropriate referrals as one of their core roles. The referrals are made to other specialist colleagues working either in the same health facility or in other facilities, and more so the higher level facilities that are specialist centres:

‘If there is a patient who needs a specialized service, then I refer that patient but basically I (do) everything involved in patient care.’ (P 02)

‘If it’s something that needs tertiary care, then we get (the patients) to the facilities that are able to handle that but (they are) still under our watch.’ (P 08)

### Leadership

Almost all the participants were involved in the management and administration in their respective workplaces. These roles were performed along with their clinical roles. Three sub-themes related to leadership emerged from the data: management and administration, teamwork and collaboration and quality assurance.

#### Management and administration

‘I am a budget (vote) controller, so when there is something to be done which needs finances I make the requisition and it is passed.’ (P 02)

‘I also work as a municipal medical officer but I am there part time and it’s mainly administrative where I monitor and supervise the health centres.’ (P 02)

‘Right now I am working as a medical superintendent. After seeing patients, I then go back to the office to do a bit of administrative work.’ (P 05)

‘I head the community health department. Ultimately even at regional referral you find yourself within management.’ (P 06)

‘I have been involved in recruitment of personnel or health workers within the region.’ (P 06)

‘I have administrative roles, 40-50% of my roles are administrative/managerial.’ (P 08)

#### Teamwork and collaboration

Family physicians identified teamwork and collaboration as important roles in their leadership. They collaborate with peers, other specialists and other health professionals:

‘We are managers of teams involved in patient care. This helps us to work together and make the team more comprehensive.’ (P 01)

‘…we work together as a team consulting with each other here and there, where we cannot (provide the care needed) we can refer.’ (P 01)

‘The things which help me do my work well is mainly having good rapport with my colleagues … such that they can give me a patient whom they think I need to assess. Working with colleagues has been very key.’ (P 02)

#### Quality assurance

In performing their leadership roles, FPs focus on quality assurance to improve the clinical outcomes of patients and the overall performance of the health system:

‘We follow guidelines on particular things especially in-patient management like in diabetes or (some of those) conditions with guidelines.’ (P 02)

‘After two years as a manager of the hospital, I joined an HIV project that was focusing on quality improvement through building capacity for health workers and health managers.’ (P 04)

‘We do a lot to improve the quality of care. ‘I have had a chance to work with health systems within the region like supervision activities and training’.’ (P 06)

‘I found myself supporting a new district to oversee their expanded program on immunisation.’ (P 06)

### Teaching and learning

Teaching and learning were identified as a strong theme in the interviews. The sub-themes included educating patients and communities, teaching students, peers and other health professionals and self-education for continuous professional development.

#### Educating patients and communities

Family physicians provide patient education during medical consultations and community outreach activities about disease prevention and health promotion:

‘I am also able to advise the family on the behavior, give advice on lifestyle and all that.’ (P 01)

‘A family physician can teach others, explain and treat the patients well and the patients get better.’ (P 05)

‘… they have a chance to get the health education they need and all the things on primary health care that would help them to live healthier lives.’ (P 08)

#### Teaching students, peers and other health professionals

Family physicians actively contribute to training other health professionals, including clinical officers, nurses and community health workers. They teach medical students, mentor young doctors and teach peers during continuous professional development sessions:

‘I did four years of health systems strengthening where I was a capacity building manager and basically my work was training, supervision and mentoring health workers in both clinical and health systems.’ (P 04)

‘I have been able to give teachings to young colleagues and even others.’ (P 06)

‘My work as a family physician entails teaching and tutoring students.’ (P 10)

#### Self-education for continuous professional development

Family physicians try to find time within their busy schedules to continue learning and develop their personal competencies:

‘I read a topic of my interest based on a patient I saw or a challenge I encountered the previous day.’ (P 06)

‘I do my personal preparations and study for teaching and clinical practice.’ (P 10)

### Challenges faced by family physicians

The themes identified as challenges faced by Ugandan FPs are listed in Table 3. Many of these challenges are interrelated, yet the following comments emerged as specific concerns.

#### Lack of identity

Most participants mentioned that patients, the general public, colleagues and students did not understand the discipline of FM. This has made it difficult for FPs to practise their specialty without a continuous sense of defence and explanation:

‘As a family physician, not so many people know about it. So many don’t know my work as a family physician. The work of a family physician is still being confused with that of a general medical officer.’ (P 05)

‘And also they see it as low level specialty because they want to be surgeon, internal medicine or obstetrics and gynaecology.’ (P 06)

‘Every day I get so many people asking me what family medicine is. I tell you, from the very junior person to the most senior people in healthy facility, people don’t know about family medicine, they don’t understand what family medicine is.’ (P 08)

‘What I have noted in family medicine even when I was in the training, I was alone. It seems family medicine is not well known to people in Uganda. They don’t differentiate family physician (medicine) from general practitioner with no postgraduate training. So, there is need for advocacy for the discipline to be known.’ (P 14)

‘Other disciplines (specialties) do not understand the role of family physician.’ (P 16)

Some participants attributed this lack of knowledge to the health system and regulatory bodies not properly positioning FM within health systems:

‘When it comes to even the regulatory bodies, they don’t appreciate for example in my training there are some things I may have learnt to do but when I do them I may be asked why did you do that, why didn’t you send to the surgeon.’ (P 01)

#### Low numbers of family physicians

Almost all participants identified challenges related to the relatively low numbers of FPs as compared to other specialists and medical officers practising in Uganda. This challenge leads to feelings of loneliness and isolation:

‘I think we just need numbers. We are very few family physicians and when you are very few your impact is not realized.’ (P 02)

‘The other challenge is that we are few. Like now I am alone working here. It is really difficult to find somebody with whom you share thoughts, plan together etc. Sometimes you feel like you are doing it alone and you are really not sure whether you are making a valid contribution.’ (P 06)

#### Conflicting roles

Participants identified conflicting roles as another common challenge. The conflicts included caring for patients and performing administrative and managerial roles simultaneously. This arose because of heavy clinical workloads and the struggles to balance clinical, leadership and teaching roles:

‘But usually I get problems especially when there are meetings at the municipality especially the technical planning meetings where all heads must attend. I find it very disturbing especially on fulfilling all the other work at the hospital.’ (P 02)

#### Unrealistic expectations

These seemed to originate not only from within the workplace but also from other stakeholders such as those linked to the ministry of health and communities. Ugandan FPs expressed concerns about their colleagues, health managers and the public expecting too much from them. This situation of FPs appears to arise from large patient numbers, their natural flexibility and ability to perform most of the roles assigned to them because of the breadth of their training:

‘The number of patients is too big yet people expect you do quality work (patient care). You see patient care needs time and if you have a lot of patients, you may not put all your time to explain some issues in depth.’ (P 02)

#### Poor organisational infrastructure

Most participants lamented about the organisational structure of the Ugandan health system that does not define a specific population to be served by the FP. This has made it difficult for FPs to practise the principles of FM and to be appropriately placed in the health system:

‘What really is a hindering factor is that we don’t have our (assigned) patients. We get patients from almost everywhere instead of maybe if you have a specific area of coverage like a village or community.’ (P 02)

Most participants expressed that they would perform better if they were appropriately placed within the healthcare delivery system:

‘The government system has no room for us, so we have no stage to perform from but in the private sector you can be seen and people will appreciate what family physicians are.’ (P 01)

‘We need a clear area where we practice family medicine.’ (P 07)

#### Lack of incentives

Participants expressed frustrations with lack of incentives mainly on career path, recognition and having a visible impact on healthcare services. These would increase the internal motivation of the FPs not only to pursue their mandate but also to elevate their status:

‘Some people want to do family medicine but they wonder after training what next, where do I go because the structure does not absorb us, has no room for us.’ (P 01)

‘There is no real career path. The financial implications of having no career path is that you end up doing multiple things to support the little salary you have.’ (P 16)

## Discussion

Most of the participants were men practising in the central region. This is explained by the male dominance of the medical profession and the Ugandan capital city being in the central region. The government is the main employer of the FPs, and then NGOs and several in private practice. Most of the participants are in clinical practice and few are in academic FM. Participants had a wide range of practice experiences, ranging from 1 to 23 years postgraduation.

Family physicians in Uganda described their roles in providing clinical care, leadership and teaching and learning. These activities included providing a variety of clinical services in outpatient clinics, emergency departments and in-patient wards. This finding reflects the realities of the Ugandan healthcare system where non-physician health personnel serve as community-based primary care providers and doctors provide services in hospital settings. These findings are similar to other studies in Sub Saharan Africa (SSA) that have found FPs mainly providing clinical services in hospitals.^20,21^

In addition to providing direct clinical services, FPs also commonly refer patients to other specialists. Many studies have documented that FPs can be trained to handle more than 80% of common health problems. Therefore, they must be skilled in recognising and referring patients who require skills outside the scope of their training.^22,23^

Family physicians identified their roles in leadership and collaboration. They provide leadership to clinical and primary care teams as well as by leading teaching and educational programmes. Family physicians may act as administrators of health facilities, medical superintendents, heads of community health departments in referral hospitals and heads of district health teams. This variety of leadership roles results from FPs not having a clearly assigned place within the government health system. As a result, some participants expressed that FPs may perform better in private settings. Some programmes have developed curricula to prepare FPs to strengthen their leadership and clinical governance skills.^24^ Therefore, findings from this study provide valuable evidence for medical educators who are responsible for developing curricula as well as healthcare planners and ministries of health in countries where FM is being established.

Family physicians are actively engaged in teaching medical students and other health professionals. Some are employed in academic institutions where their main role is to teach medical students. Even those who work in hospital settings often teach medical students because almost all medical schools in Uganda have a component of community-based education where students are placed in health facilities to learn about primary healthcare services throughout the country. Family physicians described the importance of learning throughout their careers. Despite their busy schedules, most found time for self-study. Many participants shared their desire to provide a high quality of care for their patients. They reported seeking and finding information regarding the patients’ disease conditions which they encounter in their various practices. They emphasised the importance of FPs as lifelong learners pursuing continuous professional development activities.^28,29^ They also described FPs as mentors for other health workers and young doctors. The important teaching and learning roles of FPs have been described in the CanMeds Initiative^30^ and other publications.^31,32,33^

A key challenge is described by participants related to the limited understanding of the FM discipline among the public, medical students, colleagues and key stakeholders. This lack of understanding can be attributed to FM being a relatively new discipline in Uganda and many other countries in the SSA region. Most medical schools have not yet introduced FM in their undergraduate curriculum and most medical students graduate without exposure to the discipline. Several other studies have found a lack of FM knowledge among stakeholders.^32,33,34,35^

The findings indicate that there is still a lack of a clear identity or well-defined roles for Ugandan FPs. They may practise in a variety of settings, including non-governmental organisations, heading hospitals and departments within the hospital, filling human resource gaps in delivery of health services or heading DHSs. This haphazard deployment coupled with low numbers of practitioners makes it difficult for FPs to serve as role models in medical education and in the clinical arena. The organisation of the Ugandan health system where primary care is not well defined lacks continuity, proper coordination and inadequate referral systems contributed to these challenges.

Many FPs play several roles at the same time. Therefore, they may struggle to balance clinical, administrative, managerial, teaching and mentoring roles. This heavy workload contributes to role conflicts and may lead to feelings of being overwhelmed. In addition, as most FPs do not have fellow FPs in their places of work, they may feel lonely and isolated.

## Recommendations

Family physicians should be trained to perform a wide range of skills to meet the needs of the communities that they will serve and the expectations of key stakeholders. The training of FPs should be tailored to make them ‘fit for their purpose’ and to the context in which they will practise. African FM training models should be designed to prepare FPs to address the unique challenges of African health systems.

Advocacy and stakeholder engagement will be required to ensure support for policies and funding to support the expansion of FM. Additional resources will be required to train sufficient numbers of FPs to meet the needs of communities. Family medicine training programmes will need to be expanded to recruit students, provide postgraduate training and offer continuing professional development.

The communities to be served by each primary healthcare facility should be clearly defined. Family physicians could then be placed within these facilities where they can be responsible for the health of defined populations. This would make it possible for FPs to practise the principles and philosophy of FM. This study confirms that FPs have the capacity to serve as role models and to improve the health of individuals, families and communities. However, they will be more likely to reach their full potential if they are able to practise in a supportive health system and motivating environment.

## Strengths and limitations of the study

The strengths of this study are based on interviews with a cross-sectional sample of Ugandan FPs practising from all geographical regions and clinical settings. Because the participants were selected to represent all regions, government, non-governmental and private practices, this study is likely to have identified the common roles and challenges. However, the study relied entirely on self-reports. Family physicians were not observed performing the roles they described. It is possible that some FPs may have over- or underestimated the roles they perform and their challenges. In addition, as the interview data were aggregated, we were not able to identify challenges unique to different types of practices or locations.

## Conclusion

The major roles of FPs in Uganda are similar to those of their counterparts in Africa and other parts of the world. Family physicians provide clinical care for patients, including preventive and curative services; providing leadership, management and mentorship to clinical teams; and teaching and learning. However, their roles are exercised differently as a result of lack of proper institutionalisation of FM within the Uganda health system. Family physicians in Uganda have found many places in the health system where they can contribute, but they have not yet identified a stable niche. This has contributed to the challenges they face, including lack of identity because of poor understanding of FM among the public, other health workers and health system stakeholders; conflicting roles with FPs trying to balance various roles within their workplaces; feelings of loneliness and isolation as a result of low numbers in the various workplaces; and haphazard placements within the health system. These challenges make it very difficult for FPs to fulfil their mandate consistent with the principles and philosophy of FM.
